# From Neurosurgical Planning to Histopathological Brain Tumor Characterization: Potentialities of Arcuate Fasciculus Along-Tract Diffusion Tensor Imaging Tractography Measures

**DOI:** 10.3389/fneur.2021.633209

**Published:** 2021-02-26

**Authors:** Matteo Zoli, Lia Talozzi, Matteo Martinoni, David N. Manners, Filippo Badaloni, Claudia Testa, Sofia Asioli, Micaela Mitolo, Fiorina Bartiromo, Magali Jane Rochat, Viscardo Paolo Fabbri, Carmelo Sturiale, Alfredo Conti, Raffaele Lodi, Diego Mazzatenta, Caterina Tonon

**Affiliations:** ^1^Pituitary Unit, IRCCS Istituto delle Scienze Neurologiche di Bologna, Bologna, Italy; ^2^Department of Biomedical and Neuromotor Sciences, University of Bologna, Bologna, Italy; ^3^Neurosurgery Unit, IRCCS Istituto delle Scienze Neurologiche di Bologna, Bologna, Italy; ^4^Department of Physics and Astronomy, University of Bologna, Bologna, Italy; ^5^Anatomic Pathology Unit, Azienda USL di Bologna, Bologna, Italy; ^6^Functional and Molecular Neuroimaging Unit, IRCCS Istituto delle Scienze Neurologiche di Bologna, Bologna, Italy

**Keywords:** neurosurgery, tractography, arcuate fasciculus, along-tract, gliomas grading, language network, fMRI laterality index, pre-surgical planning

## Abstract

**Background:** Tractography has been widely adopted to improve brain gliomas' surgical planning and guide their resection. This study aimed to evaluate state-of-the-art of arcuate fasciculus (AF) tractography for surgical planning and explore the role of along-tract analyses *in vivo* for characterizing tumor histopathology.

**Methods:** High angular resolution diffusion imaging (HARDI) images were acquired for nine patients with tumors located in or near language areas (age: 41 ± 14 years, mean ± standard deviation; five males) and 32 healthy volunteers (age: 39 ± 16 years; 16 males). Phonemic fluency task fMRI was acquired preoperatively for patients. AF tractography was performed using constrained spherical deconvolution diffusivity modeling and probabilistic fiber tracking. Along-tract analyses were performed, dividing the AF into 15 segments along the length of the tract defined using the Laplacian operator. For each AF segment, diffusion tensor imaging (DTI) measures were compared with those obtained in healthy controls (HCs). The hemispheric laterality index (LI) was calculated from language task fMRI activations in the frontal, parietal, and temporal lobe parcellations. Tumors were grouped into low/high grade (LG/HG).

**Results:** Four tumors were LG gliomas (one dysembryoplastic neuroepithelial tumor and three glioma grade II) and five HG gliomas (two grade III and three grade IV). For LG tumors, gross total removal was achieved in all but one case, for HG in two patients. Tractography identified the AF trajectory in all cases. Four along-tract DTI measures potentially discriminated LG and HG tumor patients (false discovery rate < 0.1): the number of abnormal MD and RD segments, median AD, and MD measures. Both a higher number of abnormal AF segments and a higher AD and MD measures were associated with HG tumor patients. Moreover, correlations (unadjusted *p* < 0.05) were found between the parietal lobe LI and the DTI measures, which discriminated between LG and HG tumor patients. In particular, a more rightward parietal lobe activation (LI < 0) correlated with a higher number of abnormal MD segments (*R* = −0.732) and RD segments (*R* = −0.724).

**Conclusions:** AF tractography allows to detect the course of the tract, favoring the safer-as-possible tumor resection. Our preliminary study shows that along-tract DTI metrics can provide useful information for differentiating LG and HG tumors during pre-surgical tumor characterization.

## Introduction

In 2014, it was argued that brain diffusion-weighted MRI tractography was not yet ready as a clinical tool (1), but recently, the synergistic collaboration of neurosurgeons, neuroradiologists, scientists, and vendors has rendered the technique suitable for clinical practice (2–8). Indeed, MRI tractography has gained a role in neuro-oncology and brain tumor surgery in both adult and pediatric patients (2–8).

Many technical limitations such as the inability of deterministic tractography to resolve kissing and crossing fibers have been overcome by the use of high angular resolution diffusion imaging (HARDI) acquisitions and innovative high-order crossing fiber models, such as constrained spherical deconvolution (9). Tractography can currently provide an accurate visualization of the spatial relationship between the intra-axial tumors, such as gliomas, and the subcortical tracts, contributing to more precise surgical planning and to guiding tumor resection intraoperatively (2–8).

Several studies have demonstrated that tractography can reliably image many white matter (WM) tracts, including the cortico-spinal tract (CST), optic radiations, and, among those involved in the language functions, the arcuate fasciculus (AF) (10–13). This latter tract is one of the most clinically relevant structures, connecting Wernicke's and Broca's areas and represents a significant portion of the superior longitudinal fasciculus (SLF), belonging to the dorsal stream, which is involved in language production and comprehension (14). The critical role of the AF is demonstrated by the interruption of speech production typically observed when the tract is electrically stimulated intraoperatively. When injured, it has been found to disrupt phonological processing and reduce speech fluency (15–18).

AF reconstruction is particularly useful for the surgery of gliomas located in the proximity of the language areas, when performed in combination with language fMRI tasks, permitting the identification of the language-eloquent cortical regions near frontal, insular, and temporal tumors in the dominant hemisphere (2–8). Indeed, it has recently been demonstrated that the adoption of this neuroimaging approach can improve surgical outcomes by reducing the risk of permanent language deficits (16).

Nevertheless, although the effectiveness of the AF tractography for guiding pre-and intraoperative resection of tumors in eloquent language areas has already been demonstrated, its ability to determine the biological nature of the tumor or assess its aggressiveness has not been thoroughly investigated (19–24). Previous studies have highlighted microstructural abnormalities in fascicles localized in proximity to a glioma in or near language or motor cortex (25). However, the absence of a reliable healthy control (HC) population for patient-specificity microstructural comparisons has led to discrepant results (19–24). Furthermore, to date, the potential role of along-tract analysis has not been tested for language eloquent area gliomas. No studies have explored the relationship between along-tract microstructural measures and the reorganization of brain activity in the presence of tumors, as determined by language task fMRI.

Our study investigates the correlation between language area tumor histopathology and AF integrity by using an along-tract analysis, as a more accurate alternative to whole-tract tractography analysis. This study aimed to demonstrate that AF tractography contributed to the safe resection of gliomas in language areas and secondarily to investigate how along-tract analyses can shed light on tumor histopathology and, in combination with fMRI, functional neuroplasticity.

## Materials and Methods

### Subjects

From August 2019 to June 2020, we recruited consecutive adult patients referred to the Functional and Molecular Neuroimaging Unit, IRCCS Istituto delle Scienze Neurologiche di Bologna (Italy), according to the following criteria: ≥18 years of age and the presence of a single primary tumor lesion in a language-related area. All patients recruited underwent a standardized MRI acquisition protocol on a 3-T scanner, and histopathological and molecular testing.

As controls, a cohort of healthy volunteers was also recruited for this study. HCs were selected from the database of the Neuroimaging Laboratory, designed to collect normative values of quantitative MR parameters for clinical and research purposes.

The study was approved by the local Ethical Committee (183/2019/OSS/AUSLBO-19027 (20/03/19), and written informed consent was obtained from all participants.

### Pre-surgery Protocol

The medical history of all patients was considered, particularly if they had already undergone surgical or adjuvant treatment for the brain tumor. Each patient underwent a complete neurological examination with a specific focus on possible language impairments, such as aphasia, anomia, paraphasia, or grammatical or syntactic mistakes, thanks to a semi-structured interview performed by a neuropsychologist. All patients and HCs were assessed for years of education and handedness dominance using the Edinburgh Handedness Inventory (EHI) (26). EHI scores between −1 and −0.5 were considered indices of left-handedness, right-handedness was defined by scores between 0.5 and 1, and scores between −0.5 and 0.5 indicated ambidextrousness. In order to ensure that patients understood and were able to execute required tasks during fMRI acquisition, they each undertook a training session for the functional paradigms.

A complete neurophysiological assessment, including somatosensorial, motor, and brainstem auditory evoked responses, was performed 24 h before surgery.

### Brain MRI Acquisition Protocol

The MRI protocol was performed using a high-field Siemens MAGNETOM Skyra 3-T MRI scanner equipped with a high-density array coil, with 64 channels and full head–neck coverage.

The MRI protocol included volumetric T1-weighted imaging based on 3D MPRAGE [176 continuous sagittal slices, 1-mm isotropic voxel, no slice gap, echo time (TE) = 2.98 ms, repetition time (TR) = 2,300 ms, Inversion Time (IT) = 900 ms, flip angle = 9°, acquisition matrix = 256 × 256, pixel bandwidth = 240 Hz, in-plane acceleration factor = 2, duration ~5 min] and volumetric fluid-attenuated inversion recovery (FLAIR) T2-weighted imaging (3D SPACE, 176 sagittal acquisition slices, 1-mm isotropic voxel, no slice gap, TE = 428 ms, TR = 5,000 ms, IT = 1,800 ms, flip angle = 120°, acquisition matrix = 256 × 256, pixel bandwidth = 780 Hz, in-plane acceleration factor = 2, duration ~5 min). In patients, volumetric T1-weighted images were also acquired after the injection of gadolinium contrast agent (0.1 mmol/kg).

For tractography analyses, a HARDI diffusion-weighted protocol was acquired with b-value = 2,000 s/mm^2^ along 64 diffusion gradient directions, and five volumes without diffusion weighting, based on a 2D single-shot echo planar imaging (EPI) sequence [87 continuous axial slices, 2-mm isotropic voxel, no slice gap, TE = 98 ms, TR = 4,300 ms, flip angle = 90°, acquisition matrix = 110 × 110, pixel bandwidth = 1,820 Hz, in-plane acceleration factor = 2, multiband acceleration factor = 3, phase encoding anterior–posterior (AP), duration ~8 min]. An additional sequence of three null b-value volumes was acquired immediately prior to the full diffusion data set, with the same acquisition geometry and timing parameters but inverted phase encoding [posterior–anterior (PA)]. The information from this sequence was used to correct EPI distortion artifacts in the diffusion-weighted scan.

In order to assess hemispheric language laterality, the neural correlates of verbal fluency were elicited via a phonemic fluency task performed during block-design functional MRI based on a 2D single-shot EPI sequence (56 continuous axial slices, 2.5-mm isotropic voxel, no slice gap, TE = 37 ms, TR = 735 ms, flip angle = 53°, acquisition matrix = 94 × 94, pixel bandwidth = 2,130 Hz, no in-plane acceleration, multiband acceleration factor = 3, phase encoding AP, duration ~5 min). The block design consisted of alternated resting and active blocks, each lasting 30 s, starting and ending with the resting condition (five resting blocks and four active task blocks in total). The active task blocks were composed of acoustic cues delivered at 5-s intervals. During resting blocks, continuous white noise was delivered. The acoustic cues were administered through MR-compatible earphones that isolated the background MRI noise. During active cycles of phonemic fluency, the acoustic cue stimulus was a letter of the alphabet, delivered every 5 s. After the presentation of the cue, subjects were prompted to covertly generate (i.e., think about) a noun starting with the given letter. Subjects were instructed to generate as many nouns as possible within the time lapse between stimuli but not to generate proper names or names of places (cities/lands/continents). During rest cycles, patients were instructed to lie quietly in the scanner without active thinking (27).

### Tumor Segmentation

The patient's tumor volume was manually segmented by LT and by an experienced neuroradiologist with more than 10 years of experience (FB) using the itk-SNAP software (http://www.itksnap.org) (28).

A multiparametric segmentation approach was used: all the voxels presenting signal intensity alterations in either the FLAIR T2-weighted or T1-weighted (with/without 0.1 mmol/kg of gadolinium contrast agent administration) images were included.

### Tractography

#### Imaging Preprocessing

Diffusion-weighted images were skull-stripped using the FSL-bet function (https://fsl.fmrib.ox.ac.uk/fsl/fslwiki). Image denoising was performed with the MRtrix3-dwidenoise function (https://www.mrtrix.org), using a principal component analysis approach. Susceptibility-related distortions in the EPI acquisition were estimated using the FSL-topup function; subsequently, a combined correction for susceptibility, eddy-current effects, and signal dropout, most commonly induced by subject movement, was performed for the FSL-topup estimates.

The FSL-dtifit function was used to model diffusivity along the spatial eigenvectors using the tensor model, obtaining the following diffusion tensor imaging (DTI) maps: fractional anisotropy (FA), mean diffusivity (MD), axial diffusivity (AD), and radial diffusivity (RD). These maps were used to assess changes in diffusivity parameters in the presence of tumor edema or infiltration.

#### Arcuate Fasciculus Tractography Pipeline

The tractography pipeline was fully automatized. High-order fiber modeling was used to evaluate crossing fibers, and a probabilistic streamline propagation approach was adopted. Regions of interest (ROIs) defined in the Montreal Neurological Institute (MNI)-152 space were non-linearly registered (FSL-fnirt function) for subject T1-weighted images. The T1-weighted images were then registered to the diffusion-weighted images using the FSL-epi_reg function, which aligns images, simultaneously correcting for distortions using gray–white intensity contrast.

To reconstruct the AF bilaterally, a previously validated seed-target approach was used, described in detail in Talozzi et al. (29). Briefly, adapting the procedure by Giorgio et al. (30), the tractography seed was defined in the MNI-152 space, located in the WM underlying the angular gyrus, anteriorly to the point where the AF begins to arch toward the temporal terminations. Symmetrical bilateral seed ROIs had a rectangular shape extending from |X| = 42 to 30, Y = −38 to −37, and Z = 20 to 34 in MNI-152 coordinate space. Tractography target ROIs were placed in both the frontal and temporal lobes, including ROIs defined by the Harvard–Oxford probabilistic atlas, thresholded at 25% of subject probability. The frontal target ROI was defined as comprising all the frontal Harvard–Oxford regions, inclusive of the precentral gyrus and precentral operculum, while the temporal target ROI comprised all the temporal Harvard–Oxford regions. Moreover, a dilatation kernel (flsmaths–dilM) was applied to include the tractography streamlines stopping just before the gray matter. A midsagittal exclusion ROI was defined at MNI-152 space X = 0.

Constrained spherical deconvolution diffusion modeling and probabilistic tractography were performed (tckgen ifod2-Mrtrix3) in native diffusion space, into which the tractography ROIs defined in MNI-152 space were non-linearly registered. Tractography results were thresholded at 10% of the maximum of connectivity within each voxel, to reduce false-positive artifactual reconstructions.

Subsequently, along-tract mapping and statistical calculations were performed in MNI-152 space. AF tractographic reconstructions and DTI maps were linearly aligned to the MNI = 152 space (FSL-flirt, allowing 12 degrees of freedom). A linear registration approach was preferred to preserve the native tract bundle geometry, allowing comparisons of patients and HCs in a common space.

#### Along-Tract Analyses

For accurate quantification of DTI maps along with the AF, a previously developed along-tract approach was applied (29). This method parameterizes the tract volume evaluating its three-dimensional mesh. In the mesh connectivity matrix, the Laplacian operator was computed; and the first Laplacian eigenvalue, which described the three-dimensional geodetic trajectory, was evaluated.

To standardize along-tract subdivision of the AF, the tract was parameterized after registration to the MNI-152 space and restricted to the compact WM core prior to its branching toward cortical areas, where intersubject variability was elevated.

MNI-152 coordinate limits were set anteriorly at y_max = 65 mm for frontal AF projections and inferiorly at z_min = 40 mm for temporal AF projections. After WM core restriction, the AF was divided into 15 equally spaced segments (29). This AF subdivision was adopted to extract along-tract DTI maps profiles within each AF segment. Along-tract analyses were performed using locally developed software written in Matlab 2019R (https://matlab.mathworks.com).

### Functional MRI Analyses

The fMRI processing pipeline was created using only FSL software. Images were skull-stripped using the FSL-bet function.

During preprocessing, motion correction was performed with the tool “motion correction of functional images using the linear image registration” (FSL-MCFLIRT) (31). Spatial smoothing was performed using a full width at half maximum (FWHM) Gaussian kernel of 5 mm.

FSL-epi_reg permitted registration between structural and functional images. High-pass filtering of task-based fMRI time series was performed with a threshold of 60 s.

Language-based fMRI data were processed using the FSL-FEAT GUI (FMRI Expert Analysis Tool) (32). Task and rest cycles in block conditions were convolved with the hemodynamic response function to generate the general linear model (GLM). For each subject, fixed-effect GLM was performed using a threshold of z ≥ 3.1, and then a cluster-extent-based thresholding was used, setting *p* < 0.05.

In order to evaluate a hemispheric laterality index (LI), the fMRI activation regions obtained were masked with bilateral ROIs to evaluate activations in selected language areas. Frontal, parietal, and temporal ROIs were extracted from the cortical Harvard–Oxford atlas:

- Frontal ROIs included the inferior frontal gyrus pars triangularis, pars opercularis, and the frontal operculum.
- Parietal ROIs included the angular gyrus and the posterior supramarginal gyrus.
- Temporal ROIs included the posterior portion of both superior and medial temporal gyri.

fMRI activations were non-linearly registered to the MNI-152 space, using the warp field defined by FSL-FEAT. fMRI activation maps registered to the MNI-152 space were thresholded at Z > 3.1 and then masked for each subject using the previously defined bilateral frontal, parietal, and temporal areas (fslmaths–mas). The number of activated fMRI voxels was evaluated within each area.

The LI was calculated according to the following formula (33):

LI = (Left – Right)/(Left + Right),

where “Left” and “Right” indicate the number of voxels activated within the left and right homologous areas, respectively.

These ROIs were investigated on the basis of a previous study that investigated the correlation between WM pathways and cortical areas related to language functions (34). While this analysis undoubtedly over-simplifies the fMRI neural activations correlating with language, it aims to robustly extract a laterality activation index in each lobe and quantify the reorganization of brain activity in the presence of tumors.

The SurfIce software (https://www.nitrc.org/plugins/mwiki/index.php/surfice:MainPage) was used for the projection of voxel-wise data onto a surface mesh and to display fMRI results in three dimensions.

### Brain Tumor Surgery

Surgery was performed in all cases with a resective aim. Intraoperative neurophysiological monitoring was used in all cases, and, when indicated, an awake setting was adopted (we opted for the sleeping–awake–sleeping technique). Anesthesia was performed consequently, avoiding the use of myorelaxant.

All surgeries were performed with neuronavigational guidance (StealthStation S8 Surgical Navigation System, MEDTRONIC, Louisville, CO, USA) provided by the co-registered data sets of morphological MRI, tractography reconstructions, and phonemic task activations.

### Post-operative Course and Follow-Up

All patients underwent an MRI with and without gadolinium contrast agent administration (0.1 mmol/kg) within 72 h since surgery to assess the extension of tumor removal. For purposes of assessment, “gross total resection” (GTR) refers to the absence of residual tumor detected by early post-operative MRI scans compared with preoperative MRI scans (i.e., with respect to any residual enhancement); “subtotal resection” refers to possible residual tumor < 10%; “partial resection” refers to possible residual tumor more than 10% (35).

Neurological and neuropsychological examination, with particular regard to language deficits, was performed at awakening at then daily for the first 5–7 days until discharge from hospital.

Surgical and medical complications were analyzed using electronic medical records. After case-by-case discussion at the tumor board multidisciplinary meeting, adjuvant treatments (radio- and chemotherapies) were started 1 month after surgery. Follow-up consisted of morphological MRI scan and neurological examination performed every 3–6 months.

### Tumor Histopathological and Molecular Characterization

Surgical specimens were formalin-fixed and paraffin-embedded (FFPE) according to routine procedures. Diagnosis was assessed by two neuropathologists (SA and VPF) according to the 2016 WHO classification of tumors of the central nervous system.

Immunohistochemistry was performed in an automated stainer (Ventana, Tucson, AZ, using Ventana purchased pre-diluted antibodies): antibodies anti-GFAP (clone EP672Y, Cell-Marquez), anti-Olig2 (clone EP112, Cell-Marquez), anti-synaptophysin (clone MRQ-40, Cell-Marquez), anti-BRAF V600E (clone VE1, Roche), anti-CD34 (clone QBEnd/10, Roche), anti-IDH1 R132H (clone H09, Dianova), anti-ATRX (polyclonal, Sigma), and anti-p53 (clone DO-7, Roche) were used. Ki67 labeling index (clone 30–9, Ventana Medical Systems Inc., Tucson, AZ, USA) was evaluated by counting at least 1,000 neoplastic cells.

Molecular analyses for IDH1 (exon 4), IDH2 (exon 4), and TERT (promoter) were performed by next-generation sequencing (NGS). Briefly, representative tissue was identified from FFPE specimens and extracted with the Quick Extract FFPE DNA Extraction Kit (Epicentre, Madison, WI, USA). Sequencing was performed using the 454 GS-Junior NGS (Roche Diagnostic, Mannheim, Germany).

The methylation status of the MGMT promoter region was assessed by MS-qLNAPCR (rapid methylation sensitive quantitative PCR assay using Locked Nucleic Acid) (36). Identification of the 1p/19q allelic status was obtained using a dual-color fluorescence *in situ* hybridization (FISH) analysis and an Olympus BX61 epifluorescence microscope: for each case, at least 100 neoplastic nuclei were counted, and the copy numbers of 1p36/1q25 and 19q13/19p25 were recorded for each nucleus (37).

Patients were stratified into two groups according to the tumor grading: low grade (LG) including dysembryoplastic neuroepithelial tumor (DNT) and gliomas grade II, and high grade (HG) including gliomas grades III and IV.

## Statistical Analyses

### Tractography

Normative ranges for along-tract AF microstructural DTI measures (FA, MD, RD, and AD) were defined, adopting the following criteria. For each of the 15 AF segments within the HC population, the median DTI value of each parameter was subtracted from the raw measure to obtain de-medianed measures, and segment outliers lying beyond three times the mean absolute deviation were removed. The normative range for each parameter was then defined by the interval bracketed by the 2.5th to 97.5th percentiles of all such segment measures, estimated from the standard deviation of distribution excluding outliers. This calculation was performed separately for the right and left AF.

For patients, within each AF segment, median DTI measures were calculated for each parameter (FA, MD, RD, and AD). AF DTI measures were considered abnormal if they laid outside the HC normative range for each DTI parameter, more precisely, when the segment median measure was less than the 2.5th percentile or greater the 97.5th percentile of the HC distribution. The total number of abnormal AF segments was counted. Additionally, the median across all segments was calculated for each parameter. For patients, outlier detection was not applied since out-of-range DTI measures are potentially a marker of pathological conditions.

### Group Comparison and Correlation Analyses

The Mann–Whitney test was used to compare LG and HG tumor patients for measures including tumor volume, along-tract DTI measures, and fMRI LI evaluated in the frontal, parietal, and temporal ROIs. The DTI-derived measures considered included the number of AF segments with decreased FA and AD, the number of AF segments with increased MD and RD, and median measures of FA, AD, MD, and RD along the AF.

An adaptive significance threshold was applied using the Benjamini–Hochberg false discovery rate (FDR) procedure to account for multiple comparisons (38). Matlab 2020 statistical and bioinformatics toolbox functions were used for these statistical analyses.

Subsequently, correlations between different DTI measures (FDR < 0.1) and fMRI LIs were calculated using the non-parametric Spearman rank, in order to evaluate the strength and significance of the structural–functional correlations (SPSS v27), again accounting for multiple comparisons using the Benjamini–Hochberg procedure (38), setting the FDR to 0.1 since in our study the number of subjects was < 20 (39).

## Results

Nine patients were recruited (age: 41 ± 14 years, mean ± standard deviation; 5 males). Tumors were located in the left hemisphere in eight cases and in the right in one. They were mainly involving the middle temporal gyrus in two cases; the inferior, middle, and superior frontal gyri in three; the insula in two; and the angular gyrus in two.

At histological examination, one was DNT, three tumors were gliomas grade II, and five were HG tumors (two grade III and three grade IV). One patient (F, 63 years old) with an anaplastic astrocytoma had been operated 13 years before for a grade II astrocytoma (Table 1).

**Table 1:** Demographic information is reported for tumor patients [low-grade tumors (LG) and high-grade tumors HG)] including education and Edinburgh Handedness Inventory (EHI) scores.

	**Age (years)**	**Sex**	**Education (years)**	**EHI**	**Location**	**Tumor volume (cm^**3**^)**	**Tumor grade**	**Histopathology**	**Molecular analysis**
**LG**									
T_L_I	21	M	13	−0.3	Left MTG	4.2	I	DNT	NA
F_L_II	30	F	13	1	Left IFG, MFG, SFG	22.8	II	Diffuse astrocytoma	IDH mutant, MGMT methylated, 1p/19q non-codelated
I_L_II	40	M	18	0.89	Left insula	122.0	II	Astrocytoma	IDH mutant, MGMT methylated
I_R_II	38	F	11	1	Right insula	43.0	II	Oligodendroglioma	IDH mutant, MGMT methylated, 1p/19q codelated
**HG**									
P_L_III	63	F	11	0.94	Left ANG	26.4	III	Anaplastic diffuse astrocytoma	IDH mutant, MGMT methylated, 1p/19q non-codelated
F_L_III	58	M	13	0.68	Left IFG, MFG, SFG	17.5	III	Anaplastic oligodendroglioma	IDH mutant, MGMT methylated, 1p/19q codelated
P_L_IV	51	M	18	0.89	Left ANG	13.1	IV	Glioblastoma	DH wild type, TERT promoter mutated, MGMT wild type
F_L_IV	41	M	19	0.78	Left IFG, MFG, SFG	57.9	IV	Glioblastoma	IDH mutant, MGMT methylated, 1p/19q non-codelated
T_L_IV	31	F	17	−0.4	Left MTG	115.7	IV	Glioblastoma	IDH mutant, MGMT methylated, 1p/19q non-codelated

*Tumor volume, grade, histopathology, and molecular analyses are also reported*.

*In the first table column, patients are labeled according to the tumor's (lobe)_(hemisphere)_(grade): frontal (F), temporal (T), parietal (P), and insular (I) lobe; right (R) and left (L) hemisphere*.

*NA, not available; MTG, middle temporal gyrus; IFG, inferior frontal gyrus; MFG, middle frontal gyrus; SFG, superior frontal gyrus; ANG, angular gyrus*.

**Figure 1:**
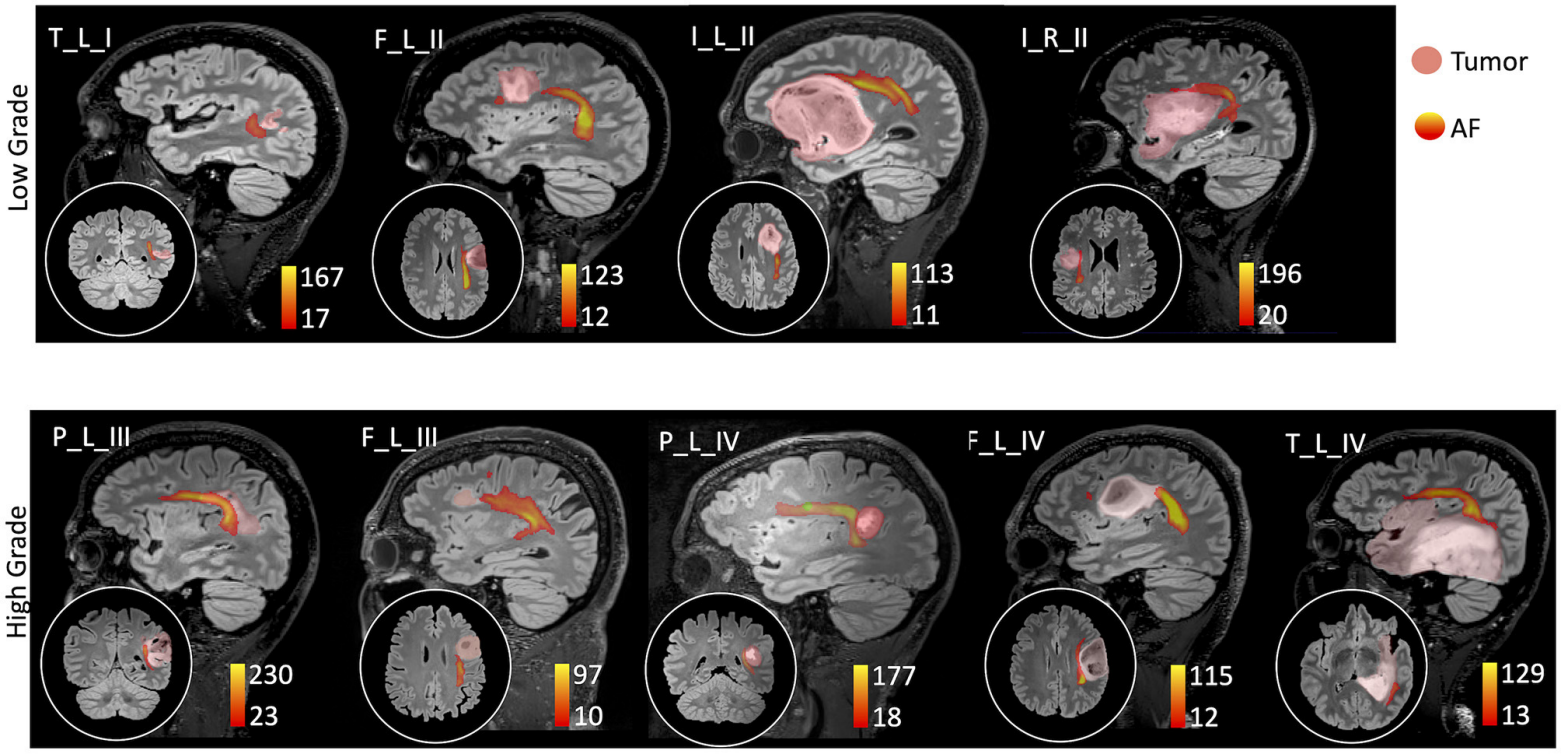
Sagittal view on the tumor side and coronal or axial focus of the spatial relationship between the tumor (pink colormap) and the arcuate fasciculus (AF) tractography (colormap red to yellow indicating increasing fiber reconstruction probability). Patients were divided according to low-grade (top panel) and high-grade (bottom panel) tumors. Patients are labeled according to the tumor's (lobe)_(hemisphere)_(grade): frontal (F), temporal (T), parietal (P), and insular (I) lobe; and right (R) and left (L) hemisphere. For patient P_L_IV, a secondary tumor component (light green) is shown within the AF course.

Thirty-two healthy volunteers (age: 39 ± 16 years, 16 males) were also recruited (Table 2). Diffusion MRI tractography identified the AF in all cases, demonstrating its spatial relationship with the tumor (Figure 1). Patients' main demographic and clinical data, and tumor characterization (location, volume, histopathology, and molecular status), are reported in Table 1, dividing patients into two subgroups corresponding to the LG and HG tumors.

**Table 2:** Demographic information is reported for the healthy control (HC) population.

**HC**	**Age (years)**	**Sex**	**Education (years)**	**EHI**
*N =* 32	39 ± 16 (18–72)	*N =* 16 male	20 ± 4 (13–30)	*N = 3* left-handedness
		*N = 1*6 female		*N = 5* ambidextrousness
				*N = 24* right-handedness

*Mean ± standard deviation (range) is shown. Edinburgh Handedness Inventory (EHI) scores between −1 and −0.5 were considered indices of left-handedness; right-handedness was defined by scores between 0.5 and 1; and scores between −0.5 and 0.5 indicated ambidextrousness*.

### Low-Grade Tumors

Three LG gliomas presented with epileptic seizures, and the DNT was an incidental finding. At hospital admission, all patients were neurologically intact, with no language impairments. The patient with DNT presented a history of dyslexia, and he referred no recent alteration in his language function.

Surgery was performed in an awake setting in two cases, and GTR was achieved in all but one case.

One patient developed a post-operative transient mild aphasia, which regressed completely in 7 days. All patients with LG tumors underwent radio- and chemotherapy. At follow-up (13 ± 1.3 months), they were alive without disease in three cases, and with a stable remnant in the other case.

### High-Grade Tumors

Seizures were the manifesting symptom in four patients with HG tumors, while in the fifth case, an asymptomatic progression was detected at scheduled MRI follow-up for LG tumor treated by surgery 13 years previously. At hospital admission, no patient presented neurological deficits.

Tumor removal was performed in awake in four of the five cases. GTR was obtained in two patients and subtotal resection in the other three. Post-operative complications consisted of one case of epileptic seizures, controlled by antiepileptic drugs. One patient presented a post-operative transient aphasia and one some semantic paraphasia, which recovered completely after 30 and 2 days, respectively.

All patients underwent radio- and chemotherapies. At follow-up (mean 13 ± 5.7 months), all patients were alive, and one locally recurred, requiring a second surgery followed by chemotherapy.

### Along-Tract Analyses

**Figure 2:**
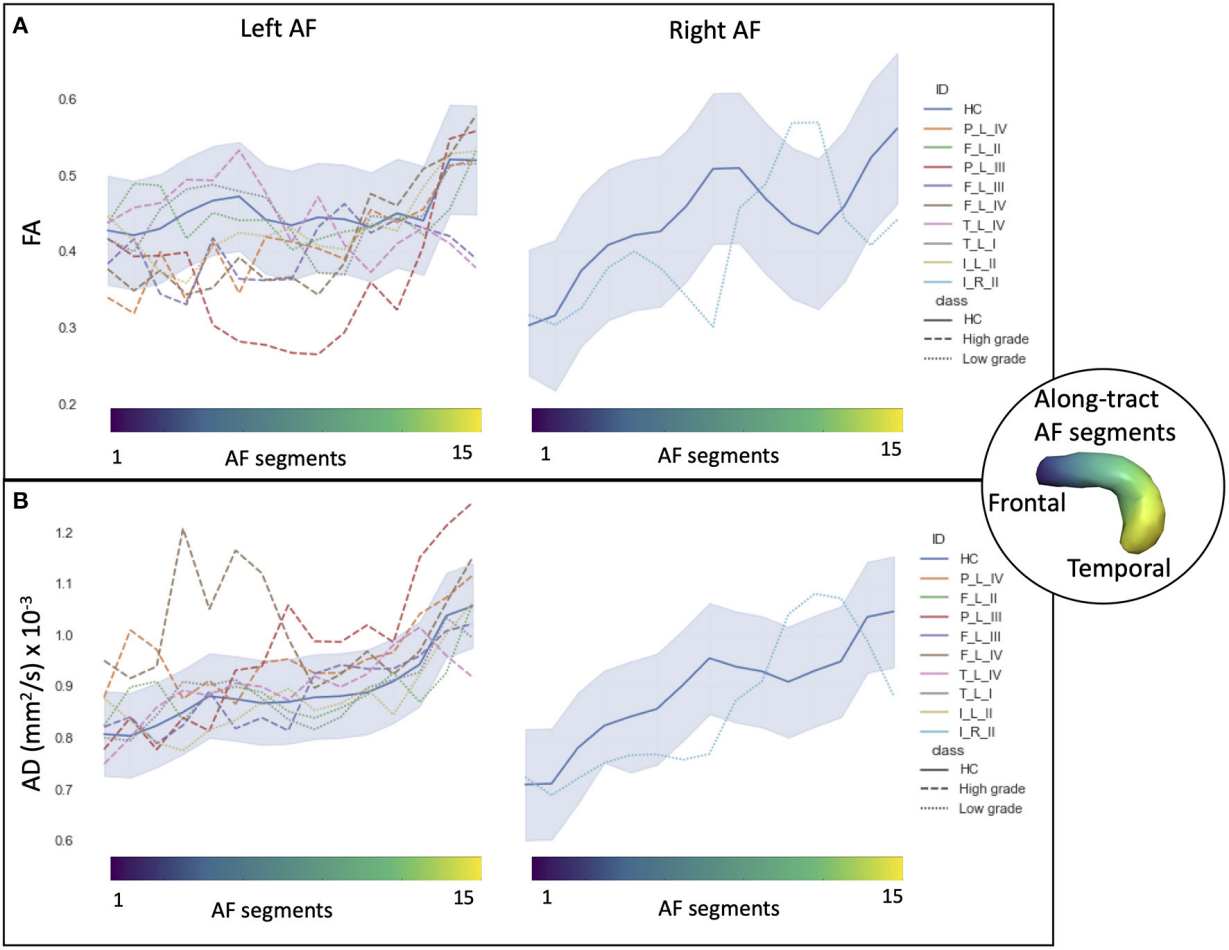
Along-tract diffusion tensor imaging (DTI) measures [**A**: fractional anisotropy (FA); **B**: axial diffusivity (AD)] of the left and right arcuate fasciculi (AFs) are shown for both the patient group and the healthy control (HC) population. The AF was divided into 15 segments, ranging from frontal (blue) to temporal (yellow) divisions. Along-tract plots were colored to identify HC normative range and patient ID. Patients are labeled according to the tumor's (lobe)_(hemisphere)_(grade): frontal (F), temporal (T), parietal (P), and insular (I) lobe; and right (R) and left (L) hemisphere. Different line styles were used for HC, high-grade, and low-grade tumor patients. The blue-shaded areas indicate the normative HC range (2.5th−97.5th percentile).

**Figure 3:**
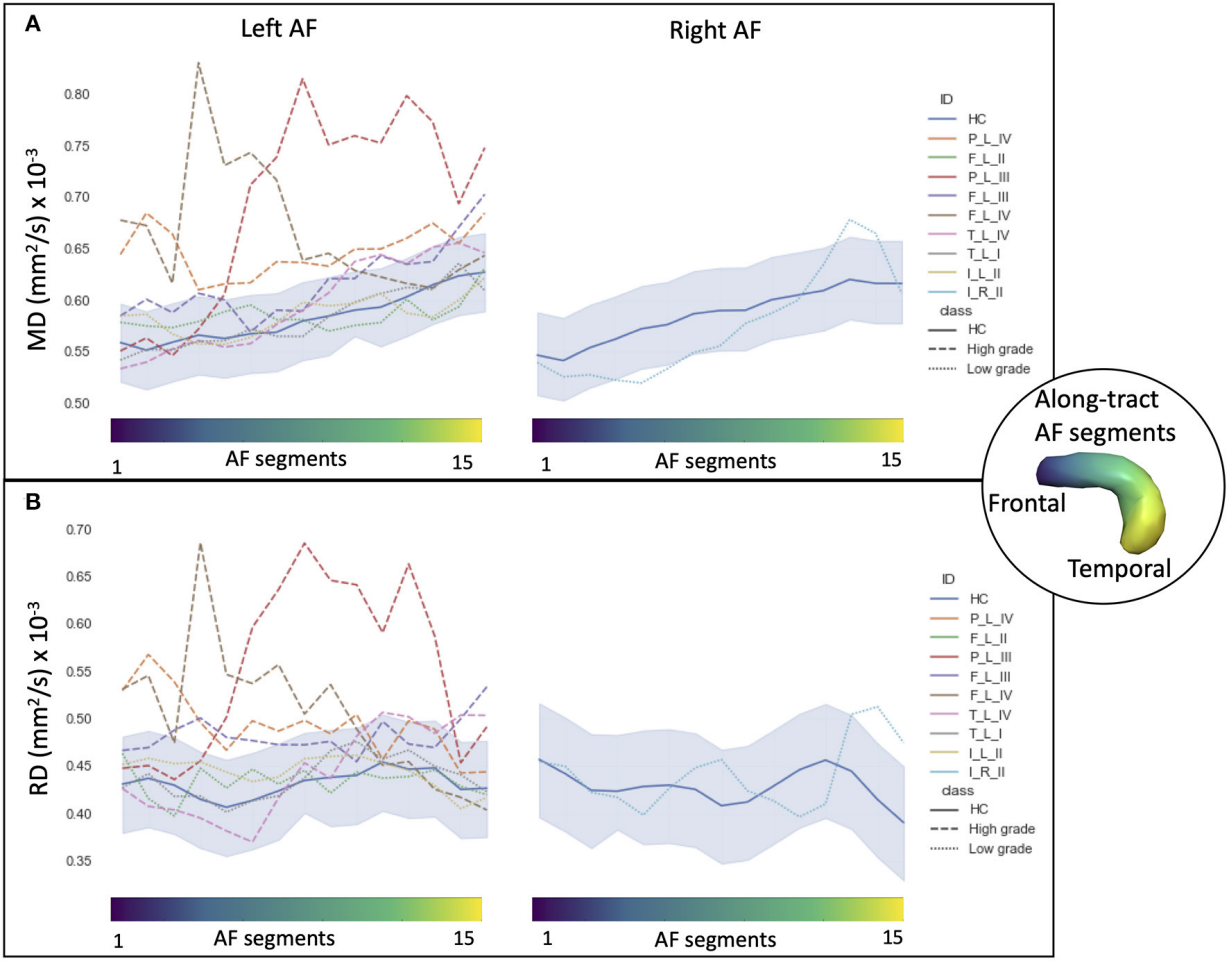
Along-tract diffusion tensor imaging (DTI) measures (**A**: mean diffusivity (MD); **B**: radial diffusivity (RD)] of the left and right arcuate fasciculi (AFs) are shown for both the patient group and the healthy control (HC) population. The AF was divided into 15 segments ranging from frontal (blue) to temporal (yellow) divisions. Along-tract plots were colored to identify HC normative range and patient ID. Patients are labeled according to the tumor's (lobe)_(hemisphere)_(grade): frontal (F), temporal (T), parietal (P), and insular (I) lobe; and right (R) and left (L) hemisphere. Different line styles were used for HC, high-grade, and low-grade tumor patients. The blue-shaded areas indicate the normative HC range (2.5th−97.5th percentile).

In HCs, the profiles of DTI along-tract measures differed between the right and left hemispheres (Figures 2, 3). Outlier detection within the distribution of each HC DTI measure was used to define a normative range. The number of values identified as outliers was between 10 (MD of left AF) and 20 (MD of right AF) out of 480 (15 segments and 32 HCs). No specific AF segment localization or control subject was more prone to producing outliers. There was no systematic bias of outliers in the upper or lower tail.

Comparing the patients' along-tract measures with HCs', no abnormal or a maximum of four abnormal segments were detected in the LG tumor group, whereas within each HG tumor patient, more than four AF abnormal segments were measured with a maximum of 14 abnormal segments (Table 3).

**Table 3:** Non-parametric comparison of low-grade (LG) and high-grade (HG) tumor patients using Mann–Whitney test, considering tumor volume, along-tract DTI measures [fractional anisotropy (FA), axial diffusivity (AD), mean diffusivity (MD), and radial diffusivity (RD)] median value and number of abnormal AF segments (#) compared with the healthy control (HC) normative distribution, and fMRI laterality index (LI).

**Measures**	**LG tumors Median (min-max)**	**HG tumors Median (min-max)**	**LG vs. HG *p-*value**	**FDR**
Tumor volume	32.9 (4.2–122)	26.4 (13–115.8)	1	1
FA # decreased segments	1.5 (0–4)	6 (2–8)	0.047^*^	0.11
Median FA	0.43 (0.40–0.45)	0.41 (0.36–0.44)	0.142	0.21
AD # decreased segments	1 (1–3)	4 (0–9)	0.306	0.37
Median AD	0.88 (0.77–0.90) mm^2^/s	0.95 (0.89–0.99) mm^2^/s	0.027^*^	**0.08**
MD # increased segments	0 (0–2)	10 (2–14)	0.017^*^	**0.07**
Median MD	0.58 (0.56–0.59) mm^2^/s	0.64 (0.59–0.74) mm^2^/s	0.014^*^	**0.07**
RD # increased segments	0 (0–3)	8 (4–10)	0.012^*^	**0.07**
Median RD	0.44 (0.43–0.45) mm^2^/s	0.50 (0.44–0.59) mm^2^/s	0.086	0.17
LI fMRI frontal	0.30 (0.02–0.86)	0.02 (−0.26 to 0.56)	0.221	0.29
LI fMRI parietal	0.37 (−0.20 to 1)	−0.18 (−0.62 to 0.18)	0.142	0.21
LI fMRI temporal	0.36 (−0.62 to 0.57)	0.33 (−0.06 to 0.75)	0.806	0.88

* *uncorrected p < 0.05. In bold are comparisons at false discovery rate (FDR) < 0.1*.

In particular, considering the FA measure, there was no clear stratification of LG and HG tumors, as both groups presented some abnormalities compared with HCs (Figure 2A): for LG tumors, a maximum of four abnormal segments, and for HG tumors, a maximum of eight. A similar unclear stratification was also detected for the AD measure (Figure 2B), for which only two HG tumors presented more than four abnormal segments.

Considering the MD and RD measures, LG and HG tumor stratifications were clearer. MD measures were normal in all segments of the left LG tumor, whereas two segments showed increased MD in the right LG, while in the AF of the HG tumors, between two and 14 abnormal segments were detected. For RD, up to three abnormal segments were found only in the right hemisphere of LG tumors, whereas a minimum of four and a maximum of 10 abnormal segments were detected in the HG patients.

### Functional MRI

Phonemic fluency activations during fMRI were evaluated both by visual inspection and by calculation of LI to assess language hemispheric dominance and agreement with the EHI handedness score.

The visual inspection of the overall task fMRI activations revealed five patients with left hemispheric dominance and four patients with a bilateral fMRI activation pattern in homologous language network regions. For six patients out of nine, fMRI activations' visual inspection was congruent with the EHI handedness scores, with a predominant activation in the left hemisphere if the patient was right-handed. The exceptions were as follows: one ambidextrous patient who presented a predominant activation on the left, and three right-handed patients showed a similar bilateral activation pattern.

**Figure 4:**
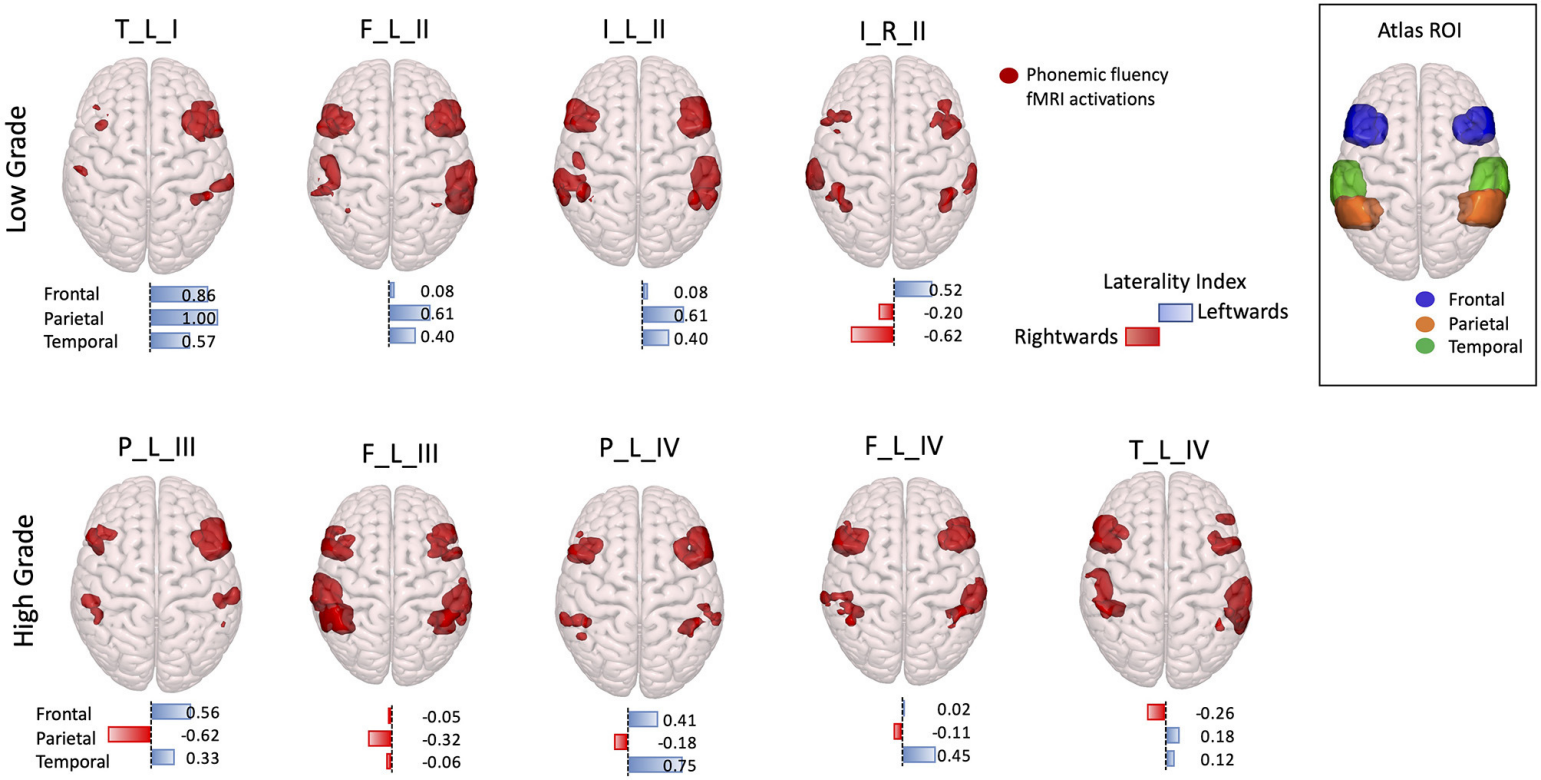
3D rendering with a superior prospective of fMRI phonemic fluency activations in the Montreal Neurological Institute (MNI)-152 brain. Activations were restricted to the frontal, parietal, and temporal regions according to regions of interest selected in the Harvard–Oxford Atlas, shown in the top right corner box. Patients were divided according to low-grade (top panel) and high-grade (bottom panel) tumors. Under each 3D-rendered brain, a horizontal laterality index bar is shown for each lobe, where the color, and orientation encode the hemisphere lateralization (red rightward and blue leftward), and the bar length gives the laterality index value. Patients are labeled according to the tumor's (lobe)_(hemisphere)_(grade): frontal (F), temporal (T), parietal (P), and insular (I) lobe; and right (R) and left (L) hemisphere.

The visual examination of fMRI activation patterns was in agreement with the LI measures, presenting predominant activations in the left hemisphere in all the LG tumor patients (LI > 0), with the exception of the right hemisphere tumor patient, whereas for the HG tumors, mixed lateralization patterns were present. In particular, within the HG tumor patients, four presented right-lateralized language parietal activations, one patient presented only frontal right-lateralized activation, and one patient presented all three lobes activations right-lateralized (Figure 4).

### Low Grade vs. High Grade Group Comparisons

In the comparison of DTI metrics across LG and HG tumor patient groups, differences were detected (at FDR = 0.1, 12 comparisons) in the number of abnormal MD and RD segments, median AD measure, and MD measures (Table 3). In particular, the number of increased MD and RD segments was higher in the HG tumor group than in the LG tumor group, and the median AD and MD measures were increased.

No differences were detected considering the tumor volume or fMRI LIs in the frontal, parietal, and temporal ROIs.

### Along-Tract Diffusion Tensor Imaging and Functional MRI Laterality Correlations

The four DTI metrics, which differed in the LG vs. HG tumor comparison, were correlated to the fMRI LIs in the frontal, parietal, and temporal ROIs.

During the phonemic fluency task, a more rightward-lateralized fMRI pattern (LI < 0) in the parietal ROIs only correlated with a higher number of abnormal MD segments (−0.732, unadjusted *p* < 0.05) and RD segments (−0.724, unadjusted *p* < 0.05) (Table 4).

**Table 4:** Spearman rank non-parametric correlations (R) between fMRI laterality index (LI) and along-tract diffusion tensor imaging (DTI) measures, which differed (FDR < 0.1) between LG and HG tumor patients [axial diffusivity (AD), mean diffusivity (MD), and radial diffusivity (RD)], are reported.

**Along-tract measures**	**LI fMRI frontal**	**LI fMRI parietal**	**LI fMRI temporal**
Median AD	0.033	−0.233	0.533
MD # increased segments	−0.034	−0.732^*^ **(***p=*****0.025, FDR=0.14)****	0.187
Median MD	−0.183	−0.55	0.317
RD # increased segments	−0.068	−0.724^*^ **(*****p=*** **0.028, FDR** **=** **0.14)**	0.17

*Number of abnormal AF segments (#), compared with the HC normative distribution, and median along-tract DTI measures were evaluated*.

* *uncorrected p < 0.05*.

*FDR, false discovery rate*.

*In bold are correlations at false discovery rate (FDR) < 0.1*.

None of the correlations survived after correcting for 12 multiple comparisons at FDR = 0.1.

### Longitudinal Case Presentation

#### Low Grade Tumor (LGC) Case 1

**Figure 5:**
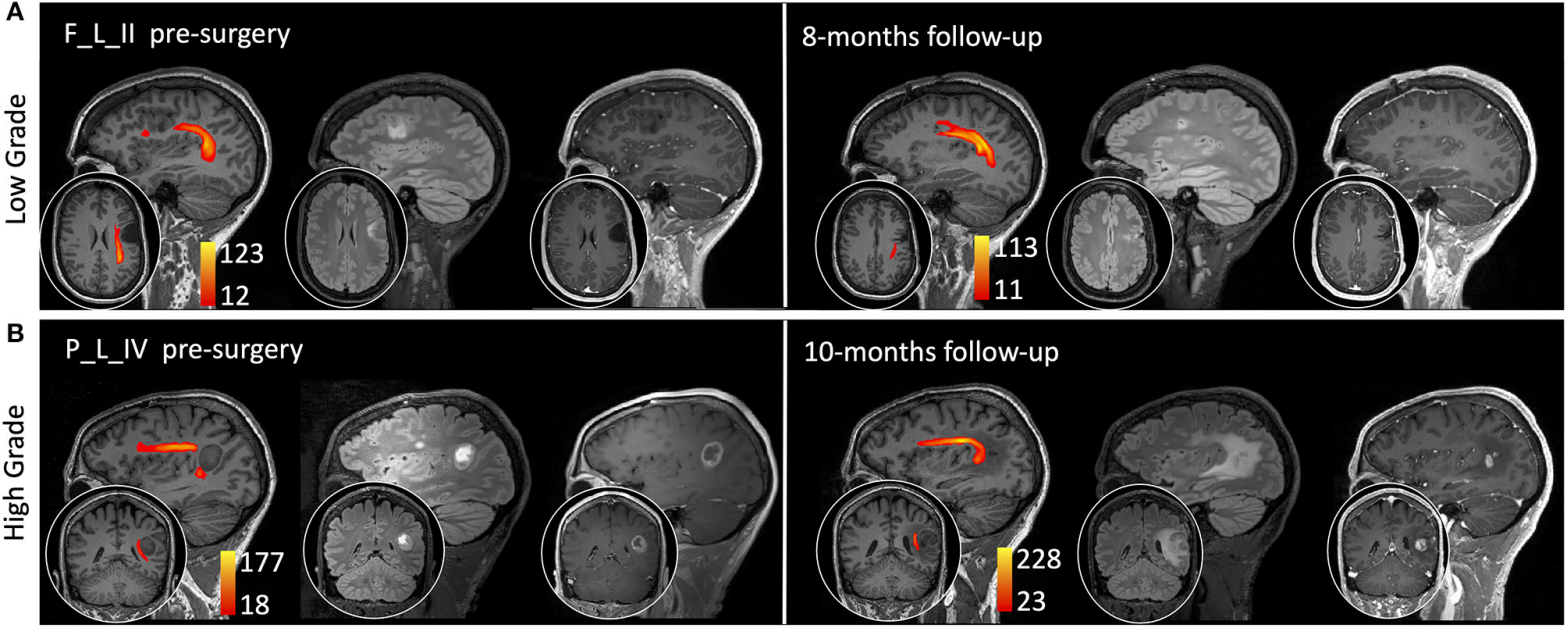
Sagittal view on the tumor side and coronal or axial focus of T1-weighted, fluid-attenuated inversion recovery (FLAIR) T2-weighted, and T1-weighted images after gadolinium administration (0.1 mm/kg). The arcuate fasciculus tractography is superimposed on T1-weighted images (colormap red to yellow indicating increasing fiber reconstruction probability). **(A)** A low-grade glioma patient pre-surgery (left side) and 8 months post-surgery (right side). **(B)** A high-grade glioma patient pre-surgery (left side) and 10 months post-surgery (right side). Patients are labeled according to the tumor's (lobe)_(hemisphere)_(grade) classification: parietal (P) and frontal (F) lobe; and left (L) hemisphere.

A 30-year-old woman came to our attention in July 2019 after she had a generalized seizure, anticipated by an episode of dysphasia. Medical history was unremarkable. Brain MRI scan demonstrated a left frontal intra-axial tumor, showing hypointensity in T1-weighted and hyperintensity in T2- and FLAIR T2-weighted images and not enhancing after gadolinium administration (Figure 5A).

**Figure 6:**
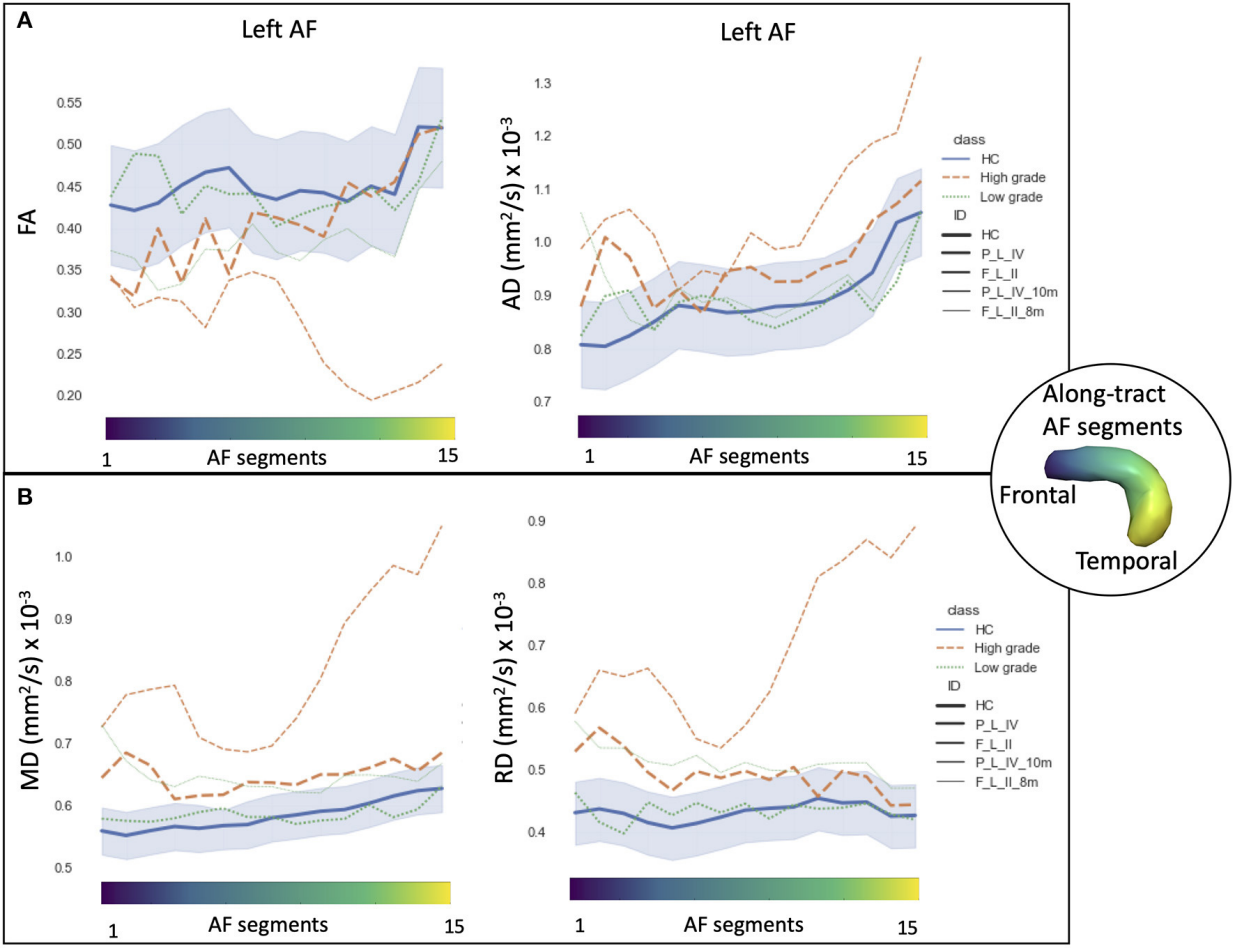
Along-tract diffusion tensor imaging (DTI) measures [**A**: fractional anisotropy (FA) and axial diffusivity (AD); **B**: mean diffusivity (MD) and radial diffusivity (RD)] of the left arcuate fasciculus (AF) are shown for the longitudinal trajectories of two patients: one high-grade tumor with a 10-month follow-up after surgery (orange dashed line) and a low-grade tumor patient with an 8-month follow-up (green dashed line). Normative healthy control (HC) DTI measures were reported: median values (blue continuous line) and normative range, from the 2.5th to 97.5th percentiles (blue-shaded area). The AF was divided into 15 segments ranging from frontal (blue) to temporal (yellow) divisions. Patients are labeled according to the tumor's (lobe)_(hemisphere)_(grade) classification: parietal (P), frontal (F), and left (L) hemispheres.

The AF tractography showed the close proximity of the tumor to the AF (Figure 1, case F_L_II). Along-tract AF DTI measures are shown in Figure 6, for both the pre-surgery and 8-month follow-up MRI scans. The fMRI activations demonstrated left hemispheric dominance (Figure 4, case F_L_II), corresponding to the right-handed dominance showed by the EHI score of 1.

Despite start of pharmacological treatment with levetiracetam 1,000 mg/day, a further seizure, with comparable semiology, re-occurred 3 months later. After an increase of the antiepileptic drug dosage, a surgical procedure was performed with awake technique. Cortical and subcortical mapping of the frontal region adjacent to the tumor was performed with direct electrical stimulation (DES), and afterwards, central debulking was performed with a Cavitron Ultrasonic Surgical Aspirator (CUSA, Integra LifeSciences, Princeton, NJ, USA).

The AF was located by the neuronavigation system, and after direct stimulation during reading, counting, and denomination of objects tasks, the portion of the tumor in its close proximity was also resected without provoking any language disturbances, achieving a gross tumor resection. The histopathological analysis confirmed the diagnosis of diffuse astrocytoma grade II, isocitrate dehydrogenase (IDH) mutant, MGMT methylated, and 1p/19q non-codelated.

Post-operative course was uneventful, and the patient was discharged home after 3 days. Three months' MRI scan confirmed the radical resection of the tumor, and at 12 months' follow-up, the patient was neurologically intact, with no recurrence of the glioma.

#### High Grade Tumor HGC Case 2

A 51-year-old man came to our attention in October 2019 for a partial epileptic seizure, consisting of an episode of aphasia with speech arrest lasting a few minutes. Medical history was unremarkable. Treatment with levetiracetam 1,000 mg/day was started, and brain MRI showed a left fronto-temporal intra-axial tumor, hypointense in T1-weighted, and hyperintense in T2- and FLAIR T2-weighted images, with peripheral enhancement after gadolinium administration (Figure 5B).

AF tractography demonstrated the close proximity of the tumor to the AF (Figure 1, case P_L_IV). Along-tract AF DTI measures are shown in Figure 6, for both the pre-surgery and 8-month follow-up MRI scans. The diagnostic suspicion was of HG. Functional MRI demonstrated a left-hemispheric dominance (Figure 4, case P_L_IV), confirmed at EHI evaluation (score 0.89), showing a right-handed dominance. LI presented a rightward activation in the parietal lobe only.

Surgery was performed with awake technique. After cortical mapping of the supramarginal and angular gyri, the tumor was initially centrally debulked. With the neuronavigation system, the location of the AF was identified, and the tumor resection in its proximity was performed until the onset of reproducible phonemic and semantic paraphasia, induced by subcortical DES. The histopathological diagnosis confirmed the nature of glioblastoma grade IV, IDH wild type, TERT promoter mutated, and MGMT wild type.

The post-operative course was characterized by semantic paraphasia, regressing in 2 days, and the patient was discharged home after 4 days. Post-operative MRI demonstrated the presence of a small remnant, and radio- and chemo-therapies with temozolomide were performed.

After 11 months, the patient presented a further epileptic seizure with dysphasia. The subsequent MRI showed progression of the remnant. Because of the excellent general conditions, a further surgery was performed in September 2020 with awake technique and 5-alanine administration. A cortical and subcortical mapping of the peritumoral region was again conducted, followed by central debulking of the tumor with CUSA. Both the AF and inferior fronto-occipital fasciculus (IFOF) was identified by the onset of phonemic and semantic paraphasia, and their location was confirmed by the neuronavigation system. WM fascicles were extensively infiltrated by the tumor (with observation of 5-alanine captation), while they appeared to be functionally intact. For this reason, resection was arrested at that level. Histopathological examination confirmed the previous diagnosis.

Post-operative course was characterized by occasional semantic and phonemic paraphasia, which progressively regressed in 2 days. The patient was discharged home after 5 days. Post-operative MRI demonstrated contrast agent enhancement at the level of the remnant. At 1-month follow-up, no language impairments were present, and further chemotherapies were planned.

## Discussion

In this pilot study, we have demonstrated that diffusion-weighted tractography of AF may be useful not only to provide accurate anatomical details for preoperative surgical planning and intraoperative cortical/subcortical brain mapping in combination with DES but also to characterize the histological grade of the tumor. Previous studies have demonstrated that decreased MD values and increased FA values show a positive correlation with the tumor cellularity (40, 41). Based on these findings, multiple studies have used DTI metrics in along-tract and perilesional regions to define brain tumor grade (42–53). Holly et al. (20, 21) described that FA and MD values were, respectively, higher and lower in perilesional regions of gliomas than in metastases. On the other hand, other studies suggest that FA increases intratumorally in gliomas (20, 21, 47, 49–51).

The possibility of differentiating between LG and HG gliomas using quantitative DTI measures is still under investigation, given the conflicting results available so far (19). As stated by Costabile et al. (4) the lack of homogeneous findings may be due to differences in diffusion-weighted acquisition protocols, pre- or post-processing methods, ROI selection, or the characteristics or size of the sample studied. Recently, Leroy et al. (19) have demonstrated that for tract fibers studied by histopathological examination after “en-bloc” resection, evaluation based on FA maps permits tract disruption to be predicted with sensitivity 89% and specificity 90%, reporting results similar to those obtained in rats (54).

Regarding AF tractography, we observed that four measures were able to discriminate (FDR = 0.1) LG and HG tumor patients: (1) median AD value, (2) number of abnormal MD segments, (3) median MD value, and (4) the number of abnormal RD segments. These results indicate the potential utility of the number of abnormal segments as a novel along-tract index, independent of tumor localization and related only to tract microstructure preservation. A future increase in the study cohort would allow us to make stronger statistical inferences. These results suggest that complementary to the role of AF tractography in the preoperative surgical planning and intraoperative brain tumor resection, the along-tract DTI measures are suitable as *in vivo* biomarkers for tract integrity. Indeed, as demonstrated by several studies, fiber tracking obtained with diffusion-weighted tractography has a high concordance rate with intraoperative findings, achieved with DES, for a large number of tracts, such as the pyramidal tract (10, 11). Remarkably, the sensitivity and specificity of diffusion-weighted tractography for SLF, IFOF, and uncinate fasciculus have been assessed at around 97–100% by Bello et al. (10). Unlike some previous studies, our tractography protocol uses constrained spherical deconvolution modeling to parameterize crossing fibers and probabilistic tracking to estimate the uncertainty in fiber propagation. These methodological steps were chosen to provide the most reliable tractographic AF reconstruction possible. Moreover, the full automated AF tractography pipeline employed allowed fiber tracking reconstruction to be standardized across subjects and allowed comparison with normative parameters derived from an HC data set.

We used the topographical information describing the spatial relationship between the glioma and the AF course to determine the surgical flap design and the operative strategy for tumor removal and to predict the risk of post-operative language disturbances. It has been demonstrated that AF preservation is correlated with a low rate of long-term language dysfunction (16). Intraoperatively, the tractography provides an optimal tool for integrate DES information in order to refine the localization of the AF during the tumor removal (55). Indeed, the current standard paradigm in brain glioma surgery involves pursuing the maximal safe resection, which means the most extensive tumor removal without affecting neurological function. Castellano et al. (56) demonstrated in their series of 73 gliomas that the most relevant parameters to achieve the maximal safe resection were the tumor volume and involvement of CST and IFOF tracts, evaluated at preoperative tractography.

Regarding functional investigations, we recorded phonemic fluency activations using fMRI to investigate tumor-related cortical plasticity by evaluating the LI in the perisylvian network. None of the frontal, parietal, temporal lobe fMRI LIs resulted significantly different in LG vs. HG tumor patients. However, when comparing the DTI measures able to discriminate LG vs. HG tumor patients and fMRI LIs, a negative correlation (uncorrected *p* < 0.05) was found between the parietal lobe LI and the number of AF abnormal MD segments (*R* = −0.732) RD segments (*R* = −0.724). Thus, these results hint at a compensatory activation of the right parietal lobe (LI < 0) and angular and supramarginal gyri, in the presence of left HG tumors characterized by a higher number of abnormal AF segments compared with the HC population.

The cortical plasticity of language functions in response to different injuries remains an open question due to the methodological difficulties and lack of sizeable patient cohorts (57). Moreover, a correspondence of structural and functional lateralization has also been reported in normal conditions (58, 59). Interestingly, Powell et al. (58) reported the most significant correlation between voxel-wise fMRI activity for verbal fluency and mean arcuate FA in the left supramarginal gyrus. However, in our study, we found a correspondence with a rightward parietal lobe activation, and MD and RD pathological tractography measures, using a newly described index, the number of abnormal AF segments compared with an HC population. This result suggests that the difference in microstructural organization compared with HCs may reflect tumor malignancy and compensatory activation in the contralateral hemisphere (60).

However, a control population for fMRI activations, equivalent to that employed for evaluating DTI measures, is needed to assess the specificity of this result to the cortical plasticity mechanism. Further limitations of the proposed fMRI analyses are the dependency of fMRI LIs to the applied threshold (Z = 3.1) as shown by Suarez et al. (61) and the mainly frontal activations obtained by administrating the phonemic fluency task (27).

We report two cases longitudinally in greater depth, from the perspective of personalized precision medicine. The along-tract DTI trajectories of an LG and HG tumors highlight the different progression rates and microstructural alterations compared with HC (Figure 6).

In the last few years, neuronavigator-integrated TMS (nTMS) has been used to identify seeding regions for DTI tractography for multiple tracts, including CTS and AF, thus improving the reliability of the DTI fiber reconstruction results (62–68). The association of these neurophysiological and neuroimaging techniques has notable advantages for reconstruction of subcortical language tracts such as the AF, which is affected by elevated intersubject anatomical variability in cortical projections (14), which can be accurately identified by nTMS, as recently shown by Giampiccolo et al. (69).

We note that the encouraging results presented in this paper are preliminary, as they were obtained in a relatively small group of patients and need to be confirmed in larger studies.

Moreover, the presence of outlier values even within the HC population suggests that there is scope for further improvement of the method of calculating along-segment DTI measures.

## Conclusion

Our pilot study suggests that the AF tractography could be considered a valid tool in the surgical planning phase and intraoperatively, guiding the subcortical eloquent regions identification. In addition, analysis of fMRI activations provides a way to delineate eloquent cortical regions. Moreover, along-tract tractography analyses can potentially characterize the histological grade of the tumor *in vivo*, as demonstrated by the higher median AD and MD values, and greater number of segments with abnormal RD or MD measures, in HG gliomas. We also found that in patients with an HG glioma, a higher number of segments with abnormal RD and MD were associated with increased compensatory language fMRI activation in the right parietal lobe contralateral to the lesion.

Our findings suggest that along-tract analysis is a useful tool in evaluating AF tractography, providing information on tumor grade and, in combination with fMRI, related cortical reorganization, thus contributing to preoperative surgical planning and longitudinal patient monitoring after surgery.

## Data Availability Statement

The raw data supporting the conclusions of this article will be made available by the authors, without undue reservation.

## Ethics Statement

The studies involving human participants were reviewed and approved by CE-AVEC. The patients/participants provided their written informed consent to participate in this study.

## Author Contributions

AC, RL, DMaz, and CTo: study design. MZ, LT, MMa, DMan, FBad, SA, MMi, FBar, MR, and VF: data collection and paper preparation. CTo, AC, RL, DMaz, and CTe: study supervision. All authors contributed to the article and approved the submitted version.

## Conflict of Interest

The authors declare that the research was conducted in the absence of any commercial or financial relationships that could be construed as a potential conflict of interest.
